# Acceptability of a remotely delivered sedentary behaviour intervention to improve sarcopenia and maintain independent living in older adults with frailty: a mixed-methods study

**DOI:** 10.1186/s12877-024-05385-4

**Published:** 2024-10-11

**Authors:** Laura J. McGowan, Angel M. Chater, Jamie H. Harper, Cherry Kilbride, Christina Victor, Marsha L. Brierley, Daniel P. Bailey

**Affiliations:** 1 NIHR Policy Research Unit in Behavioural Science – Population Health Sciences Institute, Faculty of Medical Sciences, Newcastle University, Newcastle Upon Tyne, UK; 2 Institute for Sport and Physical Activity Research, University of Bedfordshire, Polhill Avenue, Bedford, MK41 9EA UK; 3 Centre for Behaviour Change, University College London, London, WC1E 7HB UK; 4 Centre for Physical Activity in Health and Disease, College of Health, Medicine and Life Sciences, Brunel University London, Uxbridge, UK; 5 Division of Sport, Health and Exercise Sciences, Department of Life Sciences, Brunel University London, Uxbridge, UB8 3PH UK; 6 Division of Physiotherapy and Physician Associates, Department of Health Sciences, Brunel University London, Uxbridge, UB8 3PH UK; 7 Division of Global Public Health, Brunel University London, Uxbridge, UB8 3PH UK

**Keywords:** Sedentary behaviour, Frailty, Sarcopenia, Qualitative, Interviews, Intervention acceptability

## Abstract

**Background:**

Sarcopenia is a leading cause of functional decline, loss of independence, premature mortality, and frailty in older adults. Reducing and breaking up sedentary behaviour is associated with positive sarcopenia and frailty outcomes. This study aimed to explore the acceptability, engagement and experiences of a remotely delivered sedentary behaviour intervention to improve sarcopenia and independent living in older adults with frailty.

**Methods:**

This was a mixed-methods study. In-depth qualitative semi-structed interviews were conducted with a subset (*N* = 15) of participants with frailty (aged 74 ± 6 years) who had participated in the Frail-LESS (LEss Sitting and Sarcopenia in Frail older adults) intervention aimed at reducing sedentary behaviour. The interviews explored acceptability of the intervention overall and its individual components (a psychoeducation workbook, wrist-worn activity tracker, health coaching, online peer support and tailored feedback on sitting, standing and stepping). Process evaluation questionnaires with closed and scaled questions explored intervention engagement, fidelity and experiences.

**Results:**

Overall acceptability of the intervention was good with most participants perceiving the intervention to have supported them in reducing and/or breaking up their sedentary behaviour. The wrist-worn activity tracker and health coaching appeared to be the most acceptable and useful components, with high levels of engagement. There was attendance at 104 of 150 health coaching sessions offered and 92% of participants reported using the wrist-worn activity tracker. There was a mixed response regarding acceptability of, and engagement with, the psychoeducation workbook, tailored feedback, and online peer support.

**Conclusions:**

The Frail-LESS intervention had good levels of acceptability and engagement for some components. The findings of the study can inform modifications to the intervention to optimise acceptability and engagement in a future definitive randomised controlled trial.

**Trial registration:**

The trial was registered with ISRCTN (number ISRCTN17158017).

**Supplementary Information:**

The online version contains supplementary material available at 10.1186/s12877-024-05385-4.

## Background

Sarcopenia is characterised by the progressive and generalised decline in muscle mass and function associated with aging [1], which is related to an elevated risk of functional disability and dependence in activities of daily living (ADL) [2, 3]. The adverse health outcomes associated with sarcopenia include an increased risk of falls, cardiovascular disease, unplanned hospital admissions, premature death, and diminished quality of life [3]. Sarcopenia is also proposed to be a key contributing factor in the development of frailty [4]. Individuals with mild frailty have significantly increased risks of nursing home admission, unplanned hospitalisation, and mortality, with these risks being substantially higher for those with moderate and severe frailty [5]. Interventions to limit the progression of sarcopenia are, therefore, needed to help reduce the likelihood of diminished health and maintain independent living for longer.

Older adults (≥ 65 years old) represent the most sedentary age group [6], engaging in an average of 9.4 to 10.2 h of device-assessed sedentary behaviour per day [7, 8]. There is an adverse association between increasing amounts of sedentary behaviour and physical function, muscle mass and sarcopenia [9, 10]. Sedentary behaviour is also related to frailty with each additional hour of sedentary behaviour associated with 1.14 higher odds of frailty in older adults [11]. Moreover, older adults in the community with pre-frailty and frailty engage in significantly more sedentary time (86 and 73 min more per day, respectively) compared to their non-frail counterparts [12]. As well as overall sedentary time, the number of breaks in sedentary time appears to have an important role in sarcopenia and frailty-related outcomes. Older adults who engaged in less breaks in sedentary time had a higher likelihood of impairment in ADL compared with participants who accumulated more breaks [13]. Moreover, increased breaks in sitting were associated with lower odds of pre-sarcopenia [10]. Reducing and breaking up sedentary behaviour, therefore, represent promising targets to support the management of sarcopenia and frailty in older adults.

It is likely that reducing and/or breaking up sedentary behaviour is a more acceptable strategy for older adults with physical impairments, compared to engaging in moderate-to-vigorous physical activity (MVPA) [13, 14]. Indeed, systematic reviews of qualitative studies have found that older adults perceive engagement in physical activity to be incompatible with ageing [14] and perceive pain, fatigue and concerns over injury as key barriers [14, 15]. Furthermore, the adverse health associations of sedentary behaviour appear to be largely independent of MVPA [11, 13, 16, 17]. Therefore, it seems logical for healthcare interventions to target sedentary behaviour, at least initially, in older adults with frailty. Systematic review and meta-analysis evidence support the effectiveness of behaviour change interventions aimed at reducing sedentary time in community-dwelling older adults [18]. Such interventions have employed a combination of behaviour change techniques (BCTs), such as information provision, goal setting, self-monitoring, and feedback on behaviour [18]. However, it remains uncertain whether interventions designed for non-frail older adults are acceptable and effective for individuals facing frailty-related challenges, such as reduced physical function and difficulties in ADL [4, 12].

A pre-post design pilot study of a sedentary behaviour intervention (‘Stomp Out [Prolonged] Sitting’) involving a small sample of older adults with frailty (*n* = 23) demonstrated improvements in the number of breaks in sedentary time, physical function, and quality of life [19]. This 14-week intervention incorporated motivational interviewing, feedback on physical function, and a wearable activity tracker providing real-time feedback and prompts. However, the intervention did not change daily sedentary time and lacked comparison with a usual care control group [19]. In a randomised controlled trial (RCT), an intervention focusing on increasing standing exercises in older adults with frailty (*N* = 43), supplemented by health education and telephone consultations, resulted in a significant reduction of 30 min per day in sedentary time over 16 weeks, although this effect was not sustained at 30-day follow-up [20]. Previous sedentary behaviour interventions have failed to evaluate health and wellbeing outcomes, thereby limiting conclusions regarding their clinical significance.

An RCT demonstrated the feasibility and safety of delivering and evaluating a novel multicomponent intervention, Frail-LESS (LEss Sitting and Sarcopenia in Frail older adults) [21], which targeted reductions and breaks in sedentary behaviour with the overall goal of improving sarcopenia and independent living in older adults with frailty. There were also indications of efficacy for improving sarcopenia and wellbeing [21]. The Frail-LESS intervention development and content has been described in full previously [22]. Frail-LESS was developed using the Behaviour Change Wheel [23] and used the BCT taxonomy v1 to specify content [24]. The intervention comprised of a psychoeducation workbook, tailored feedback, a wearable activity tracker, health coaching, and peer support. A novel aspect of the intervention was it being delivered entirely remotely. This was viewed as important for community-dwelling older adults living with frailty, particularly those encountering challenges with ADL, who may have difficulty leaving their homes and traveling independently. These difficulties are associated with increased sedentary time in older adults [25] and could affect their ability to engage sufficiently in interventions. A further novelty was individual tailoring, as older adults frequently identified this as an appropriate intervention mechanism for reducing sedentary behaviour [15]. The use of technology was also cited as a suitable intervention strategy [15], hence the inclusion of a wearable activity tracker to monitor inactive time and prompt regular movement. A process evaluation, in line with the UK Medical Research Council process evaluation framework [26], explored the acceptability and experiences of the Frail-LESS intervention in older adults with frailty, in addition to intervention adherence and fidelity. The outcomes of this evaluation are reported in this paper.

## Methods

### Design

The process evaluation for this study used a complementarity mixed-methods design, whereby qualitative and quantitative methods were used to measure similar and different aspects of a phenomenon with data from one set of findings being expanded upon by data from another to produce an enriched, elaborated account of the situation [27, 28]. We used an explanatory sequential design (quantitative followed by qualitative), with equal weight given to each method [29]. The quantitative component comprised questionnaires with closed and scaled questions to explore intervention engagement, fidelity and experiences; the qualitative component comprised one-to-one semi-structured interviews to explore experiences and acceptability of the intervention, in-depth. The study protocol has been previously published in full [22] and was conducted in accordance with the Declaration of Helsinki. Ethical approval was obtained from the Berkshire B National Health Service Research Ethics Committee (27,051-NHS-Jan/2021–310). Written informed consent was provided by participants.

### Participants

Participants were community-dwelling older adults, aged ≥ 65 years, with very mild or mild frailty who had participated in the Frail-LESS intervention [22]. All intervention participants were invited to complete the process evaluation questionnaires and a subset took part in the interviews. For full details of participant eligibility and recruitment methods, see the published trial protocol [22].

### The Frail-LESS intervention

The Frail-LESS intervention development process and content is described in full previously [22]. The intervention was delivered remotely, comprising of five components: a psychoeducation workbook (Additional file 1), tailored feedback (Additional file 2), wearable activity tracker (wrist-worn Garmin Vivofit, Garmin Ltd. Kansas, U.S.), health coaching, and peer support; these are described in Table 1. The intervention lasted 6 months for each participant and was delivered between November 2021 and December 2022 (during the COVID-19 pandemic when self-isolation restrictions were in place for individuals with a positive COVID-19 test). The workbook, tailored feedback and wearable activity tracker were provided to participants by email and post by the research team. Health coaching sessions were delivered by individuals with backgrounds in behaviour change and psychology. Peer support sessions were facilitated by a member of the research team.

**Table 1:** Description of the Frail-LESS intervention components

Component	Description
*Psychoeducation workbook*	A psychoeducation workbook was provided (Additional file 1), which included information tailored to older adults around the health risks of excess sitting, the potential benefits of reducing and breaking up sitting, goal setting, action planning, ideas for reducing sitting, and problem solving
*Tailored feedback*	At each measurement timepoint participants received a personalised feedback sheet on their sitting, standing, stepping and breaks in sitting based on data collected using an activPAL device (PAL Technologies, Glasgow, Scotland) (see example in Additional file 2)
*Wearable activity tracker*	Participants received a wrist-worn Garmin Vivofit activity tracker to use during the intervention. The device provided feedback on inactive time with a ‘move bar’ that fills up across the display in response to prolonged periods of inactivity with an audible alert. Ambulating for several minutes is needed to prevent it from filling and to reset the bar. Feedback on steps and energy expenditure is also provided, along with a function for setting daily step goals
*Health coaching*	Health coaching was provided on a one-to-one basis using motivational interviewing [30] and the G.R.O.W. (Goal, Reality, Options, Will/way forward) model of health coaching [31]. A COM-B (Capability, Opportunity, Motivation – Behaviour) real-time analysis was used during the session with delivery of tailored BCTs guided by motivational interviewing principles. The health coaches had backgrounds in psychology and behaviour change and received training for this intervention by A.M.C and D.P.B. Health coaching sessions took place within five days from intervention start, followed by sessions at approximately 2, 6, 12 and 18 weeks. Each session was delivered via video call or telephone
*Peer support*	Online peer support meetings were organised and facilitated by the research team on a monthly basis that participants had the option of joining. The meetings centred on participants discussing their experiences with sedentary behaviour and the Frail-LESS intervention, barriers and problem solving in relation to sitting less, participants giving each other social support, and trying out different non-sedentary activities. There was also a WhatsApp group set up by a Frail-LESS participant that participants could use to interact with one another

### Data collection

#### Quantitative

Questionnaires were completed at 3 and 6 months, asking participants whether they had engaged with each of the intervention components and to rate their experiences with each component throughout the intervention up to that respective timepoint (Additional file 3). Attendance at each of the health coaching and peer support sessions that were available to participants was also recorded. Closed questions asked participants to indicate whether they did or did not engage with each intervention component, in addition to rating their experiences and opinions of each component on 5-point Likert scales e.g. from 1 “Strongly agree” to 2 “Strongly disagree”.

#### Qualitative

One-to-one semi-structured interviews were conducted either via telephone or video call, according to participant preference. Interviews took place 0–3 months following completion of the 6-month questionnaire. The topic guide (Additional file 4) included questions relating to understanding of the intervention, motivations for reducing sedentary behaviour, and experiences with the intervention components. Interviews were transcribed verbatim using transcription software (Otter AI, Otter.ai, Inc., Mountain View, CA, USA) and subsequently checked for accuracy by the research team.

### Data analysis

Data analysis for the quantitative and qualitative study components were conducted separately, with the results of each interpreted collectively within the discussion of study findings [32].

#### Quantitative

Questionnaire and adherence data (as an indicator of fidelity) were analysed descriptively using means and standard deviations, frequencies and percentages. Attendance rate for the health coaching sessions was calculated as the number of sessions attended / number of sessions available × 100. Attendance rate for the peer support sessions was calculated as the number of participants who attended each session / number of participants enrolled into the intervention.

#### Qualitative

The Framework Method [33] was used to analyse the qualitative data in line with guidance for use in healthcare research [34]. The Theoretical Framework of Acceptability (TFA) [35] was used as the overarching deductive framework. This framework explores intervention acceptability in the context of affective attitude, burden, ethicality, intervention coherence, opportunity costs, perceived effectiveness, and self-efficacy (see definition of these terms in the results section). Inductive coding within the TFA framework allowed for data-driven insights and contextual factors to be explored in more detail.

Analysis followed five main stages: *1. Familiarisation:* Reading and re-reading of transcripts and listening to audio-recordings; *2. Coding:* Line-by-line coding of a selection of transcripts, which were then mapped to the TFA domains – this resulted in a working thematic framework of TFA categories and inductively generated codes within each TFA category. Additional categories and codes were created where data did not fit within the TFA domains, and the framework was refined as more transcripts were coded; *3. Indexing:* The thematic framework was then applied systematically to all transcripts, with each code in the framework assigned a number for easy identification; *4. Charting:* Separate matrices were created for each category, and data were summarised into cells according to respective codes (presented in separate columns) and cases (i.e. individual participants, presented on separate rows); *(5) Interpretation:* Matrices were reviewed with connections and discrepancies identified within and between codes and cases, facilitating determination of the key issues and meaning within the final themes. Discussions were held within the research team throughout the analysis process to aid interpretation and ensure alternative perspectives were considered, thus enhancing rigor and trustworthiness of findings.

### Reflexivity

The lead author is a female researcher and Chartered Psychologist. Her PhD (Psychology) thesis focused on the topic of sedentary behaviour in older adults. She has considerable training and experience of qualitative interviewing with a range of participant groups. She delivers methodological teaching to undergraduate and postgraduate students on qualitative methods, mixed-methods, and theoretical approaches to behaviour change intervention development and evaluation.

## Results

### Quantitative findings

#### Intervention adherence, fidelity, and experiences

Questionnaire data was available for between 17 and 24 participants depending on the intervention component and study timepoint (Tables 2, 3, 4, 5). Five health coaching sessions were available for each of the 30 participants enrolled into the intervention. There was acceptable fidelity for the health coaching sessions with an overall attendance rate of 69% (attendance at 104 of the 150 sessions available). Attendance rate was 70%, 73%, 77%, 67% and 60% at intervention start, 2, 6, 12 and 18 weeks, respectively. For the peer support sessions, fidelity was low with an average attendance rate of 20%. Of the participants that completed the relevant process evaluation questionnaire items, 29% self-reported that they completed the psychoeducation workbook in full and 42% partially completed it (Table 2), demonstrating low levels of fidelity. There was high fidelity for the wearable activity tracker with 92% of participants reporting using the Garmin watch. There was mixed engagement with the online peer support group and tailored activPAL feedback sheet, with 46% and 58% of participants reporting that they engaged with these components, respectively (Table 2).

**Table 2:** Self-reported engagement with the Frail-LESS intervention components

		**3-Months**		**6-Months**	
		**(*n* = 24)**		**(*n* = 23)**	
		n	%	n	%
Workbook	*Yes, all of it*	7	29%	-	-
	*Yes, partially*	10	42%	-	-
	*No*	7	29%	-	-
Garmin vivofit	*Yes*	22	92%	17	74%
	*No*	2	8%	6	26%
Frail-LESS peer support group	*Yes*	11	46%	12	52%
	*No*	13	54%	11	48%
Health coach sessions	*Yes*	23	96%	20	87%
	*No*	1	4%	3	13%
activPAL feedback sheet	*Yes*	14	58%	17	74%
	*No*	10	42%	6	26%
Use of other tools/devices	*Yes*	11	46%	15	65%
	*No*	13	54%	8	35%

% calculated as number of responses / number of participants that fully completed this set of questionnaire items at each timepoint × 100

**Table 3:** Experiences with the psychoeducation workbook

		**3-Months**	
		**(*n* = 17)**	
		n	%
The level of the education workbook was appropriate	*Strongly agree*	5	29%
	*Agree*	12	71%
	*Neither agree nor disagree*	0	0%
	*Disagree*	0	0%
	*Strongly disagree*	0	0%
The amount of information was appropriate	*Strongly agree*	5	29%
	*Agree*	11	65%
	*Neither agree nor disagree*	0	0%
	*Disagree*	1	6%
	*Strongly disagree*	0	0%
The education workbook increased my awareness of the health risks of too much sitting	*Strongly agree*	8	47%
	*Agree*	5	29%
	*Neither agree nor disagree*	4	24%
	*Disagree*	0	0%
	*Strongly disagree*	0	0%
Overall, the workbook motivated me to make a change to the time that I spend sitting	*Strongly agree*	10	59%
	*Agree*	3	17.5%
	*Neither agree nor disagree*	3	17.5%
	*Disagree*	1	6%
	*Strongly disagree*	0	0%

% calculated as number of responses / number of participants that fully completed this set of questionnaire items × 100

**Table 4:** Engagement and experiences with the Garmin Vivofit activity tracker

		**3-Months**		**6-Months**	
		*n* = 22		*n* = 17	
		n / mean	% / SD	n / mean	% / SD
In the first month, how often did you use the Garmin device?	*Every day*	20	90%	-	-
	*A few times per week*	1	4.5%	-	-
	*Once a week*	0	0%	-	-
	*Infrequently*	1	4.5%	-	-
On average, in the past 3 months, on how many days each week did you use the Garmin device?	*Every day*	20	91%	13	76%
	*6 days per week*	0	0%	2	12%
	*5 days per week*	0	0%	2	12%
	*4 days per week*	0	0%	0	0%
	*3 days per week*	0	0%	0	0%
	*2 days per week*	1	4.5%	0	0%
	*1 day per week*	1	4.5%	0	0%
How useful was the Garmin device for reminding you to break up your sitting? (*5* = *Extremely useful, 1* = *Not at all useful)*		3.4	1.5	3.9	1.1
The Garmin device encouraged me to reduce the time I spend sitting	*Strongly agree*	9	41%	4	23%
	*Agree*	6	27%	11	65%
	*Neither agree nor disagree*	6	27%	1	6%
	*Disagree*	0	0%	1	6%
	*Strongly disagree*	1	4.5%	0	0%

% calculated as number of responses / number of participants that fully completed this set of questionnaire items × 100

**Table 5:** Experiences with the health coaching

		**3-Months**		**6-Months**	
		*n* = 23		*n* = 20	
		n / mean	% / SD	n / mean	% / SD
How many sessions have you had with a health coach?	*One*	1	4%	1	5%
	*Two*	9	39%	0	0%
	*Three*	8	35%	6	30%
	*Four*	4	17%	5	25%
	*Five*	1	4%	8	40%
How useful did you find the health coaching for helping you to reduce your sitting? (*5* = *Extremely useful, 1* = *Not at all useful)*		4.1	0.95	4.3	0.91
The health coaching sessions helped encourage me to reduce the time I spend sitting	*Strongly agree*	8	35%	8	40%
	*Agree*	10	43%	10	50%
	*Neither agree nor disagree*	2	9%	2	10%
	*Disagree*	3	13%	0	0%
	*Strongly disagree*	0	0%	0	0%

% calculated as number of responses / number of participants that fully completed this set of questionnaire items × 100

The content of the workbook was considered appropriate and it was useful for increasing awareness and motivation around sitting for most participants (Table 3). There was high engagement with the Garmin watch with the majority of participants using it daily and reporting that it encouraged less sitting (Table 4). The usefulness of the health coaching was rated highly and most participants found that these sessions helped reduce their sitting (Table 5). Engagement and experiences with the online peer support group was mixed (Additional file 5) with participants reporting that they communicated infrequently with other people in the group. Some participants did report, though, that the peer support group encouraged them to sit less. The usefulness of the tailored activPAL feedback sheet was rated highly, with 78% and 70% of participants reporting that it encouraged them to sit less at the 3 and 6-month timepoints, respectively (Additional file 5).

### Qualitative findings

Fifteen participants took part in the interviews. An overview of participant characteristics is shown in (Table 6).

**Table 6:** Participant characteristics (*N* = 15)

Characteristic	n (%)
Age (years)	
Mean (range)	75 (67–86)
Sex	
Female	11 (73.3)
Male	4 (26.6)
Ethnicity	
Asian or Asian British	1 (6.7)
Black, African, Caribbean or Black British	1 (6.7)
Other	1 (6.7)
White British	12 (80.0)
Employment status	
Disabled and unable to work	1 (6.7)
Employed full-time	0 (0)
Employed part-time	1 (6.7)
Retired	13 (86.7)
Unemployed looking for work	0 (0)
Home type	
Bungalow	0 (0)
Flat	7 (46.7)
House	7 (46.7)
Sheltered Accommodation	1 (6.7)

#### Intervention acceptability

**Fig. 1:**
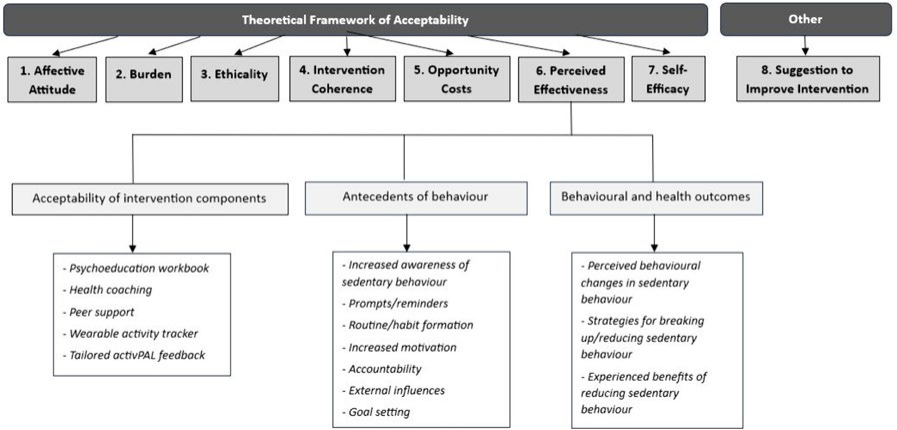
Overview of themes and sub-themes

Themes and sub-themes relating to the seven TFA domains (1. Affective Attitude; 2. Burden; 3. Ethicality; 4. Intervention Coherence; 5. Opportunity Costs; 6. Perceived Effectiveness; 7. Self-Efficacy) from the qualitative analysis are presented in Fig. 1. A final theme relating to specific ways in which participants felt the intervention could be improved is also described, resulting in a total of eight themes.

A large amount of discussion within interviews related to how participants perceived the intervention to have influenced their behaviour, including the acceptability of each intervention component, the specific drivers (i.e. antecedents) of behavioural change, the actual behavioural changes made and the experienced outcomes of these changes. Therefore, the theme ‘Perceived Effectiveness’ (theme 6) represents a large focus of our presented findings and has been broken down into multiple sub-themes.

**Affective attitude (how an individual feels about the intervention)**

Most participants held positive general attitudes towards the intervention, perceiving it to be a useful and enjoyable experience.

*“I found it a largely positive experience.” (P01 [participant ID])*

“*I think Frail-LESS has done a lot of good for me.” (P05)*

“*It [Frail-LESS] helps. I'm just liking it put it that way.” (P03)*

The Frail-LESS study was considered a valuable opportunity to receive support as an older person.

*“When I had the opportunity to have support to get active and go get back to where I was, I just grabbed it.” (P11)*

Participants discussed physical health reasons as personal motivations to reduce their sedentary behaviour, including retaining muscle mass, keeping joints healthy, weight management, reducing cholesterol, and general health and longevity*.* Retaining independence was discussed by some as an overarching motivation to maintain their physical health and mobility.

*“I do definitely want to retain activity in my life because so often people give up and I don't want to give up.” (P10)*

*“Because I think keeping mobile and being able to move around without difficulty is the very first precept for older people keeping well.” (P12)*

Some participants also discussed more affective reasons for reducing sedentary behaviour, including improving their mental and emotional health and wellbeing. This was particularly pertinent to one participant who was recently bereaved.

*“Some of it [motivation] is mental health because of the grief, and so I'm using activities to keep sane if you like.” (P11)*

Though most participants were generally positive in their attitudes, one participant held a negative perception of the Frail-LESS intervention, which was mainly due to feeling that breaking up periods of sedentary behaviour was disruptive to the enjoyment of sedentary activities.

*“If you have a line between plus five and minus five, then I come out at minus one with my agreement that this [the intervention] was a successful thing […] a lot of the pleasure I have in life is sedentary.” (P14)*

**Burden (the perceived amount of effort that is required to participate in the intervention)**

Participants experienced little burden of the intervention in itself, however, some found engaging in intervention components that required use of technology problematic. This pertained mainly to the Garmin watch, with some participants finding it frustrating and difficult to get connected.

*“There was nothing that I found negative about it, except the watch… trying to overcome the technology side of it.” (P03)*

*“I couldn't connect, which is a disappointment … I've tried and can't. It is a frustration to me.” (P14)*

Further, some participants described how they struggled to engage with the online peer support group due to issues with eyesight and hearing.

*“My hearing is not too good. So being in something with a lot of different people. I don't think I would have heard whatever they're saying.” (P04)*

*“I didn't get involved with that [peer support group], again, partly because of my eyesight.” (P03)*

**Ethicality (the extent to which the intervention has a good fit with an individual’s value system)**

In line with the intervention aims, participants generally agreed that reducing sedentary behaviour and incorporating more movement into their daily routines was beneficial to health.

*“So, I know I've got to keep moving. I knew that before. But now it's been confirmed.” (P01)*

*“It's [the intervention] really highlighted to me the importance of keeping mobile […] I had no idea before I started it, how important it was to keep significantly mobile throughout the day.” (P12)*

One participant alluded to the wider economic context of the intervention in terms of reducing demands on the healthcare system through improved health and well-being in older adulthood.

*“[It] makes my demand less on the National Health Service […] if you reduce that, and it's quite feasible, economically.” (P05)*

Some participants discussed how the intervention aims aligned with their personal goals of being more active.

*“I always wanted to do more [activity], but the Frail-LESS programme created that categorisation for me.” (P05)*

For some, the ethicality aspect of the intervention aims was in line with their self-identity. For example, one participant described themselves as someone who *‘can’t be sitting down too long… I can't just be glued to my seat… that's not me’* (P01). Another participant commented that, as they perceived themselves to be a ‘non-exerciser’, the Frail-LESS programme had been particularly beneficial to them.

*“I was never a person to exercise or do anything like that. So [the intervention] did help.” (P04)*

Another participant who had identified as being active commented how the intervention made them realise they were not as active as they had thought.

*“It really did make me sit up because I honestly thought I was a busy person, active, but I wasn't. I wasn't as active as I ought to be.” (P11)*

**Intervention coherence (the extent to which the participant understands the intervention, and how the intervention works)**

Participants generally held a good understanding of the intervention aims and methods.

*“Basically, your aim is to improve the lives of older people. So yeah, the lesson is keep moving if you want to be healthy.” (P01)*

Many participants spoke of reducing sedentary time as a distinct behaviour, rather than equating this to physical inactivity. One participant noted that reducing sedentary behaviour was a valuable goal over and above engaging in physical activity.

*“That was something that I learned from this system, that if I do, for example, go for a walk then the rest of the day I can sit… No, I don't do that [now].” (P05)*

Other participants noted that movement of the arms was non-sedentary even if they were not otherwise moving around.

*“it's not cheating, because it's moving anyway.” (P01)*

*“I have also extended that to when I'm standing, or I'm sitting, I try to move my hands or you know, do things, which moves other muscles also.” (P05)*

Reducing sedentary behaviour was deemed more appropriate for their generation than engaging in higher intensity physical activity.

*“The speed [of physical activity] is somewhat inappropriate for my generation. But it's the controlled sometimes slow work which the bottom line of which is getting up from my seat but more importantly, sitting down onto my seat in a controlled manner.” (P14)*

**Opportunity costs (the extent to which benefits, profits, or values must be given up to engage in an intervention)**

There was little discussion regarding loss of opportunities. However, one participant did note that frequently breaking up sitting resulted in a loss of enjoyment of activities and hobbies they usually took pleasure in.

*“Sitting has its own purpose. I'm currently doing a fair bit of crochet […] I enjoy reading […] getting up and down in the middle of those activities would reduce your enjoyment of them.” (P14)*

Some participants noted that lack of time and having competing commitments and priorities impeded on their engagement in some intervention components, particularly the peer support group.

*“People forgot about it [peer support group], they could have been doing other things. It's summer they could be going on holiday. Some people are still working. So yeah, that went on the back burner, unfortunately.” (P02)*

The issue of competing priorities and time constraints was also referenced in relation to engagement with the intervention workbook.

*“I didn't [use it]. I remember looking at it the first day and then after that, you know, I had so much on my mind, doing [other] things.” (P05)*

**Perceived effectiveness (the extent to which the intervention is likely to achieve its purpose)**

Themes relating to perceived effectiveness were generated for each component of the intervention (i.e. psychoeducation workbook, health coaching, peer support, wearable activity tracker, and tailored activPAL feedback) in addition to antecedents of behaviour and behavioural and health outcomes.

***Effectiveness/acceptability of intervention components***

**Psychoeducation workbook**

There was a mixed response to the psychoeducation workbook component of the intervention. Some participants found this useful in terms of ideas and prompts for reducing and breaking up sitting, and felt the way the workbook was presented was accessible.

*“It was a very good booklet. And the way it was described, it was so easy to do it […] I became very hopeful when I read it.” (P05)*

However, other participants expressed that they did not engage with this component of the intervention, with one stating they *‘looked at and forgot about it’* (P01).

*“I remember getting that right at the beginning, early days and filling it out. And it's in a drawer somewhere now […] I looked at it once and that was that.” (P08)*

Some felt that the workbook was not sufficiently tailored to older adults’ lives, and questioned the appropriateness of some of the content.

*“Maybe the questions might have been wrong or a bit too vague because it's more like one little booklet fits a lot of people maybe.” (P09)*

**Health coaching**

Many participants stated that they found the sessions with their health coach enjoyable, and for some, these sessions were useful in exchanging ideas and increasing motivation for reducing sedentary behaviour.

*“They're asking different questions for different information […] it's an exchange of ideas between the two people.” (P02)*

*“They were very helpful. She was very good and very nice. And I enjoyed having a chat with her. And she was very encouraging, which is always nice.” (P03)*

However, not all participants felt that the sessions were helpful.

*“It never registered with me put it that way. It didn't help. We discussed at the time, what I did or things like that. But once I put the phone down, I just went back to doing my ordinary duties and things like that.” (P04)*

**Peer support**

There appeared to be a general lack of engagement with the peer support group, with participants often citing competing priorities and commitments as reasons for non-attendance/engagement.

*“I think I joined it once. You know, most of the time I'm so busy. I didn't have the time really.” (P04)*

For those who did attend at least some sessions, there was split opinion over their usefulness. Some found the exchange of ideas and strategies to overcome barriers useful.

*“Seeing the problems that you've got health wise and the way that they were overcoming their problems – it’s the transference of ideas between different people with different attitudes.” (P02)*

*“I've enjoyed your [peer support] sessions […] getting us to open up and to share. They've been really good.” (P08)*

By contrast, one participant described being a private person as a reason for not engaging.

*“I didn't do it and I couldn't do it for one good reason – I do not talk about my issues with people I don't know. It was literally a privacy thing.” (P12)*

**Wearable activity tracker**

For many participants, the Garmin watch was a useful intervention component, with some perceiving it to have the biggest impact on their behaviour.

*“The watch was what really assisted me in every way, it's what really pushed me to move and do little things.” (P04)*

Participants found self-monitoring of their activity through the watch useful in particular, and some even purchased their own watch following the intervention.

*“And I also bought a watch like the one I got from the Frail-LESS, and I'm keeping track of [my activity] as well.” (P04)*

The red line that appeared on the watch screen after long periods of inactivity served as a useful prompt to stand up and move around.

*“So, it does it does help me if I see that when I see the red line. I realised ‘Oh, time to get up’. So, it does work. It does help.” (P04)*

Some participants had issues using the watch, indicating that digital literacy may have been a barrier. Issues with connection to the accompanying smartphone app led some participants to stop using the watch altogether.

*“It didn't work for me. It took me a fair while to even get it connected to my PC. And then it didn't speak to it... I gave it a few weeks and then stopped wearing it.” (P03)*

Some participants felt frustrated that the watch did not count some of their movement and activity.

*“And I do gardening as well […] you hoover your house, you know, it’s quite exhausting but it doesn't […] register on your watch.” (P01)*

**Tailored activPAL feedback**

Participants found the personalised activPAL feedback on sitting, standing and stepping ‘interesting’ and were surprised by the level of detail the device provided.

*“It's surprising what it can tell what information it can carry. So that made me feel even more conscious about behaviour.” (P09)*

However, there was contrast as to how much this influenced their sedentary behaviour.

*“They gave me information, but I don't think they made a specific difference. So, it was more informative, but not motivational.” (P03)*

*“This shock thing was the pie charts when I realized it was my activity, or rather inactivity […] I thought, I’ve got to improve this.” (P11)*

Further, some participants expressed that they did not fully understand the feedback.

*“Can't say I read it and understood it. It was just different portions, yellows, greens […] I really didn't understand it.” (P04)*

***Antecedents of behaviour***

In this theme, participants described the factors and processes which appeared to trigger them to make changes to their sedentary behaviour.

**Increased awareness of sedentary behaviour**

The majority of participants described increased reflection and awareness of their sedentary behaviour due to the intervention, which appeared to facilitate reduced sitting.

*“It made me more aware of what I was doing wrong […] more conscious of things that I should be doing, including not sitting down for too long.” (P09)*

**Prompts/reminders**

Prompts and reminders were discussed as antecedents of behaviour change. Prompts sometimes occurred naturally within participants’ own consciousness as a result of increased awareness of their sedentary behaviour. They also came directly from the Garmin watch’s inactivity alerts and the watch being worn serving as a reminder itself.

*“But I would get up… it kind of prompted me to get up once I saw the red bar.” (P04)*

**Routine/habit formation**

Changes to sedentary behaviour became embedded in some participants’ daily lives as routines and habit.

*“It's become part of life. So, you just carry on, like good habits. Just carry on doing them.” (P02)*

The importance of having routines in older adulthood, and how this helped engagement in non-sedentary activity, was emphasised by one participant.

*“Routine is important […] make sure that you do a task in the morning, like maybe clean the kitchen floor, or clean the windows or do something that will physically exert your body. And do a routine during the week. You're giving yourself a routine that you wouldn't necessarily have if you were retired, and you were alone […] Oh, I absolutely have it imprinted in me now.” (P12)*

**Increased motivation (due to the intervention)**

Increased motivation within the self was discussed in relation to changes in sedentary behaviour.

*“I found it very motivating and kept it in my mind that I had to do certain things which I may have not [previously] carried out.” (P07)*

**Accountability**

Some participants felt that being part of the Frail-LESS intervention made them accountable for reducing their sedentary behaviour.

*“It did make me want to move more even though you couldn't know exactly what I was doing. It made me more conscious or feel more guilty about not doing it possibly.” (P09)*

Some participants felt accountability towards family and friends who were aware of their participation in the study.

*“If anybody messages me, yes, I've been out. I've been and done a mile or five miles or whatever, I've got people who are checking up on me for those reasons.” (P11)*

For one participant, this sense of accountability also extended to having accountability to oneself.

*“It was like having a supervisor, internal supervisor, ‘Right. You've been sitting long enough. Now you need to get up and do something’ […] Knowing that you are accountable, I think that helps a lot.” (P12)*

**External influences**

Participants spoke of external influences on their behaviour, mostly related to the impact of seasonality and weather. In general, pleasant weather conditions and climate facilitated increased movement and less sitting. Poor weather conditions were perceived to compromise participants’ ability to maintain their behavioural changes.

*“At the moment, it's very good, because the weather is good. So, I can get out. I can do things […] bad weather would stop it. I don't like going out when it's too cold, or when it's pouring down with rain and I'm getting soaked to the skin.” (P02)*

The impact of the COVID-19 pandemic was also discussed, with participants perceiving reductions in physical activity due to lockdown restrictions and avoidance of going out in public.

*“It was reduced initially by lockdown […] the furthest I've walked since before the first lockdown is across the road to church.” (P03)*

*“COVID is still about now. I'm aware that it's still about. […] I certainly don't want to be in public spaces.” (P10)*

Some participants also commented on how their home environments impacted on their ability to engage in non-sedentary activity, both in terms of physical indoor space, and the wider local environment.

*“I am very lucky in the type of house I have, right, so there was no question of them [family] being disturbed by anything I was doing. […] If I was living in a smaller house that may have been a concern.” (P07)*

*“I'm moving to a place that is a lot more rural than where I'm coming from, and so the availability of nice walks is a lot better.” (P12)*

**Goal setting**

Some participants engaged in goal setting to support changes in their sedentary behaviour, with one participant using the Garmin watch to set stepping goals.

*“Getting my goals in […] I found that was a good incentive, I downloaded the app and paired it up [with the watch].” (P11)*

One participant commented that setting short-term goals (using the workbook) was relatively straight-forward, but perceived long-term goal setting to be more difficult.

*“The first part of it is the very easy part. It's the second part, the long term, is slightly more difficult when you first get that pack given to you.” (P02)*

***Behavioural and health outcomes***

Participants described their perceived changes in sedentary behaviour, the specific strategies and ways in which this was enacted, and the health outcomes they experienced as a result of their behavioural changes.

**Perceived behavioural changes in sedentary behaviour/impact of intervention**

Many participants reported that they had reduced their sedentary behaviour due to the intervention and perceived the intervention to have had a positive impact.

*“I was certainly making more effort to move around and to stand up.” (P03)*

One participant commented that, having received their activPAL feedback, their change in sedentary behaviour had not been as pronounced as they had initially thought.

*“When I look at my feedback, which I have in front of me, it is not all that marvellous […] I didn't improve it as much as I should have done.” (P10)*

**Strategies for breaking up/reducing sedentary behaviour**

Strategies discussed to break up and/or reduce sedentary behaviour included incorporating more movement around the house (e.g. making cups of tea, doing more housework and gardening, standing whilst cooking, standing up more frequently whilst watching television, extending periods of time spent standing/walking, and moving arms more) and incorporating movement into typically sedentary activities like dancing whilst using the telephone.

“When I was cooking, I would try to stand more - I always have a stool there. So, I've tried to stand for longer.” (P03)

“Even when I'm on the phone […] and they put the [hold] music, it's not boring for me anymore. I dance with that, stand up and dance with that tune.” (P05)

One participant had reduced travel-related sedentary time by walking instead of driving for short journeys.

“I've really tried to stop using the car for short journeys […] just as easy to walk.” (P12)

**Experienced benefits of reducing sedentary behaviour (physical and mental)**

Participants reported various benefits of reducing their sedentary behaviour, some of which related to physical well-being.

“As I do more now, my knees are hurting less, definitely.” (P01)

“I feel stronger and I feel fitter […] and I have lost some weight.” (P11)

“The physiological ones [benefits] of keeping my heart going and my blood flow going, keeping my body active.” (P14)

Some participants also discussed the benefits to their emotional and mental well-being.

“If I go out walking I always feel better after it.” (P02)

Further, some participants described the feeling of accomplishment at reducing their sedentary behaviour.

“I can sit there and smile at myself and think, ‘yeah, it worked’.” (P11)

**Self-efficacy (the participant’s confidence that they can perform the behaviour[s] required to participate in the intervention)**

Participants’ belief in their capabilities to reduce sedentary behaviour was influenced by the impact of physical limitations and existing health conditions.

* “I developed another foot ulcer. So, I haven't been able to increase my mobility.” (P03)*

*“I find it frustrating, basically, because I can't do what I used to do donkey's years ago.” (P02)*

Arthritis was commonly cited as a reason for physical limitations and pain, having a negative impact upon participants’ confidence to engage in the intervention. Attitudes towards ageing played a role in self-efficacy, with one participant commenting on the importance of not letting their identity as an older adult restrict what they perceived they could do.

“I try to avoid this idea that you're old therefore you're decrepit. I think if you're old, you can still be active and alert and all these things. […] So, this sort of study will make you think well, yes, there are still things you can do […] it's the attitude towards how you look on being older.” (P10)

**Suggestions to improve the intervention**

Some participants made suggestions for how the Frail-LESS intervention could be improved, and these have been considered and presented outside of the TFA framework. Many felt that having in-person group meetings would facilitate the positive effects of the intervention, including fostering a sense of community and togetherness.

“To come out and walk and go somewhere and be together and develop that sense of, you know, like a team […] if we have a core of people who know each other more and are more in connection, then that core can create a good impact.” (P05)

It was also suggested that participants could be put in contact with others who lived in the same locality to facilitate in-person social meet-ups, suggesting a place-based approach may be a promising avenue for future iterations of the intervention.

“Tell them about the people that are really close locally to each other [..] to meet with for a coffee or something […] create a Frail-LESS community in different addresses.” (P05)

It was suggested that the workbook content could be more tailored to the lives of older adults, with one participant suggesting that including ideas from participants themselves would be beneficial.

“And it could be made more reflective of what an older person's life is actually like, because there's very little motivation to do anything. If you're living alone, and you're retired, you can stay in bed until midday […] so perhaps saying, routine is important.” (P12)

“I think in future issues, you may also include the suggestions of different participants […] that people have added different reasons and excuses for walking and standing.” (P05)

One participant suggested that the ideas and suggestions within the workbook should be things that can be easily embedded into peoples’ daily routines, and should be purposeful activities within themselves, e.g. doing household chores.

“Rather than just do exercise, make the exercise part of your everyday living so that you can just keep on doing it. And you see the benefit of it. If you've got nice sparkly windows or a nice clean kitchen floor at the end of it.” (P12)

Further explanation of the activPAL data was suggested by one participant to help facilitate better understanding compared to graphical outputs.

“I don't know these graphs and things like that. I think it should just be put into plain English. ‘Your sitting time has been 12 hours this week […] at your last visit it was this’ instead of the graphs.” (P04)

Some participants suggested having more frequent check-ins with health coaches, which appeared to be linked to having accountability to someone else.

“Maybe more frequent with the health coach […] I just think that if I'd been checked, perhaps on a weekly basis, I might have been a slightly better at keeping it keeping going.” (P10)

“That [health coaching] was quite spaced out […] but it was really good, because again, it's accountability.” (P12)

It was suggested by some participants that individual capabilities could be better accounted for within the intervention, possibly by having distinct capability groups.

“I think I think lumping us all together is quite difficult […] because I can't compare myself with someone who hasn't been out of the house nine months and lives on their own and has got rheumatoid arthritis.” (P08)

“Older people have got different things wrong with us […] and some have got more capabilities. Some are more athletic than others in the first place […] ideally, I suppose we should have been sorted into our own capabilities.” (P09)

## Discussion

This paper explored the acceptability, adherence and experiences of a remotely delivered intervention to reduce sedentary behaviour and improve sarcopenia and independent living in older adults with frailty. The intervention was well-received overall, with most participants holding positive attitudes towards the intervention and perceiving it to have minimal burden and costs to opportunities. The wrist-worn activity tracker and health coaching sessions appeared to be the most acceptable intervention components and had high engagement. Acceptability and engagement with the other components were mixed. Many participants perceived the intervention to have had a positive impact on their sedentary behaviour through increased awareness of sedentary time, increased motivation to reduce sedentary behaviour, embedding more movement into daily routines, and having accountability to oneself and to others. Prompts and reminders to stand up and move more, as well as goal setting, were considered particularly effective techniques to support reductions in sedentary behaviour.

The wrist-worn activity tracker may have had good acceptability due to its inclusion of self-regulation strategies, such as self-monitoring, prompts and reminders, feedback on behaviour, and goal setting. Delivering these strategies using wearable activity trackers has previously been found to facilitate increased physical activity and reduced sedentary behaviour [36]. Wrist-worn activity trackers, in particular, have been found to be acceptable due to their comfort and ease of use in older populations [37, 38], and can be easily integrated into day-to-day life due to their resemblance to an everyday wrist-watch [39]. These factors could account for the high engagement with this component of the Frail-LESS intervention. Wearable activity trackers that support self-regulation of sedentary behaviour, therefore, appear to be acceptable and have a positive impact on sedentary behaviour in older adults with frailty. That said, having an in-person group tutorial at the start of the intervention to support setting up the wearable tracker’s accompanying smartphone application may further enhance acceptability, as many participants had difficulty with this.

The acceptability and high engagement with the health coaching sessions may be explained by their use of motivational interviewing techniques. Motivational interviewing is an acceptable and effective component of lifestyle interventions [40] and was found to be acceptable in a previous intervention aimed at breaking up sedentary behaviour in older adults with frailty [19]. Further, the health coaching sessions helped to facilitate ongoing commitment to the intervention through support and encouragement. This aligns with previous literature demonstrating the importance of encouragement and social support in the acceptability of reducing sedentary behaviour in older adults [41].

Acceptability and engagement with the psychoeducation workbook, tailored feedback and peer support was mixed. The workbook was useful for some participants in terms of ideas and prompts for breaking up sitting, but others did not take much interest in it or felt the content was not tailored to older adults. Education around sedentary behaviour has been used effectively in previous interventions [18], but often in the form of individual or group sessions where further tailoring to the participants may have been possible [20]. Some participants did not fully understand the tailored feedback provided in the Frail-LESS intervention. Having individual or group sessions to go through the tailored feedback, helping participants to better understand it, may be more acceptable and was suggested as a possible improvement to the intervention. It was also suggested that having in-person peer support would have improved acceptability by fostering a sense of community and enhancing social support. The opportunity for in-person support was limited due to restrictions in place to minimise the spread of COVID-19 and the intention for Frail-LESS to be delivered remotely. In-person support might also exclude individuals who are unable to leave their home and/or travel to other locations. A future intervention could, therefore, provide different options for online or in-person peer support and education to provide greater flexibility for personal preference to enhance acceptability and engagement.

The antecedents of behaviour identified in the present intervention align with other research demonstrating the acceptability of sedentary behaviour interventions in older adults. An intervention in generally healthy older adults that incorporated an information booklet with tips for displacing sitting with light activity and forming activity habits led to raised awareness of the negative consequences of sedentary behaviour, increased awareness of participants’ own engagement in sedentary behaviour, and participants feeling that the intervention served as a ‘spur to action’ [42]. The current study found that changes to sedentary behaviour became embedded in participants’ daily lives as routines and habit, which links to the wider literature on the habit-formation model of behaviour change [43]. Although participants were able to form new habits, physical limitations and pain linked to health conditions, such as arthritis, had a negative impact on participants’ confidence to engage in the intervention. Matei et al. [42] also identified pre-existing health conditions as a key barrier to intervention engagement. The present study extends previous knowledge, showing that barriers relating to physical limitations and existing health conditions in older adults with frailty can be overcome in the formation of new habits to reduce and break up sitting.

Holding positive attitudes towards ageing positively impacted participants’ self-efficacy to engage with the Frail-LESS intervention. This is congruent with previous literature that identified perceived control over the impacts of ageing and self-identity as an older adult as important influences on older adults’ confidence to reduce sedentary behaviour [41]. This is also reflective of literature on influences on older adults’ physical activity, whereby the evolving socio-cultural ageing discourse, and negative attitudes of society towards the ageing population, appear to impact on older adults’ confidence to be physically active [14, 44]. These findings suggest that intrapersonal and societal attitudes towards ageing are important influences on older adults’ self-efficacy for optimal levels of both sedentary behaviour and physical activity.

Previous studies have identified that older adults often equate sedentary behaviour to physical inactivity, lack knowledge of the distinct health consequences of sedentary behaviour, and struggle to conceptualise ways to reduce sedentary behaviour that do not involve engaging in exercise [15, 45]. Participants in the present study generally appeared to understand the distinction between sedentary behaviour and physical inactivity and explained a number of effective strategies they used to reduce or break up sedentary time, primarily involving more standing and movement around the home. This suggests that the Frail-LESS intervention employed effective methods for supporting older adults in understanding the distinct health risks associated with sedentary behaviour, helping them to utilise strategies for reducing sitting that were not ‘exercise’ orientated.

Strengths of this study include the mixed-methods design, allowing for an in-depth understanding of the acceptability, experiences, engagement and fidelity of the Frail-LESS intervention. This has generated data that can be used to modify and strengthen the intervention before conducting a definitive RCT. Further, use of the TFA [35] in the qualitative analysis ensured that the findings were relevant in the context of multiple facets of intervention acceptability. Collection and analysis of qualitative data involved multiple members of the research team with a variety of disciplinary backgrounds (e.g. health psychology, sports and exercise psychology, behavioural science, public health), therefore ensuring a wide scope of interpretation of the data and enhancing rigor and trustworthiness of our findings. Limitations of the study include the disproportionate representation of White British and female participants in the sample, limiting generalisability to other ethnic groups and males. Furthermore, as the intervention was delivered remotely, the generalisability of the findings to interventions involving in-person delivery may be limited. Lastly, fidelity in relation to the quality of the health coaching sessions (e.g. delivery of the intended BCTs) was not evaluated, which should be considered for inclusion in a future trial.

## Conclusions

The Frail-LESS intervention, which targets reductions in sedentary behaviour in older adults with frailty, had good levels of acceptability. There was high engagement with the wrist-worn activity tracker and health coaching, with mixed acceptability and engagement for behaviour change strategies delivered through a psychoeducation workbook, tailored feedback, and peer support. The findings of this study can inform modifications to the intervention to optimise acceptability and engagement with each of the intervention components in a definitive RCT, in addition to informing interventions more widely that aim to reduce sedentary behaviour in older adults with frailty.

## Supplementary Information

Additional file 1.

Additional file 2.

Additional file 3.

Additional file 4.

Additional file 5.

## Data Availability

The datasets supporting the conclusions of this article are available in Figshare, 10.17633/rd.brunel.26028892.v1. The raw qualitative data (transcripts) are not publicly available due to privacy restrictions. Further detail on the qualitative data and analysis that supports the findings of this study are available upon request to the corresponding author.

## Abbreviations

ADL: Activities of daily living
BCTs: Behaviour change techniques
Frail-LESS: LEss Sitting and Sarcopenia in Frail older adults
MVPA: Moderate-to-vigorous physical activity
RCT: Randomised controlled trial
TFA: Theoretical Framework of Acceptability
