# Supplementary material for: Acceptability of a remotely delivered sedentary behaviour intervention to improve sarcopenia and maintain independent living in older adults with frailty: a mixed-methods study
Source: BMC Geriatr. 2024 Oct 11;24:820. doi: 10.1186/s12877-024-05385-4 (PMC11468285; doi:10.1186/s12877-024-05385-4)
Supplement: Supplementary file 3 — Additional file 3. [file 12877_2024_5385_MOESM3_ESM.docx]

**Additional File 3.** Process evaluation questionnaires for intervention participants

**INTERVENTION EVALUATION – 3 MONTHS**

Frail-LESS study

**Section 1: Education session/workbook**

As part of the Frail-LESS programme, you were provided with a paper-based education workbook.

1. Did you complete the education workbook? Yes all of it ☐ Yes partially ☐ No ☐

If **no or partially competed**, please explain why you did not fully complete the workbook here: …………………………………………………………………………………………………………………………………………………………………………………………………………………………………………………………………………………………………………………………………………………………………………………………………………………………………………………………………………………………………………………………………………………………………………………………………………………………………………………………………………

If you answered **no** to the previous question, **please move onto the next section**. Only complete the next question if you answered Yes all of it or Yes partially.

1. Please tick the box which best matches your overall opinion of the Frail-LESS education workbook:

|  | Strongly disagree | Disagree | Neither disagree or agree | Agree | Strongly Agree |
| --- | --- | --- | --- | --- | --- |
| The level of the education workbook was appropriate (i.e., the information provided was easy to understand) |  |  |  |  |  |
| The amount of information was appropriate |  |  |  |  |  |
| The education workbook increased my awareness of the health risks of too much sitting |  |  |  |  |  |
| Overall, the workbook motivated me to make a change to the time that I spend sitting |  |  |  |  |  |

What were the key messages that you took away from the education workbook:

1……………………………………………………………………………………………………………………………………………………………………………………………………………………………………………………………………………………………………………………..

2.……………………………………………………………………………………………………………………………………………………………………………………………………………………………………………………………………………………………………………………..

3.……………………………………………………………………………………………………………………………………………………………………………………………………………………………………………………………………………………………………………………..

**Section 2: Wearable device feedback**

You were provided with a Garmin Vivofit wearable device to help you to track your sitting time and provide prompts to break up sitting.

1. Did you use the Garmin device given to you? Yes ☐ No ☐

If **YES go to Q2**, if **NO** please use this space to tell us the reasons why you did not use it

………………………………………………………………………………………………………………………………………………………………………………………………………………………………………………………………………………………………………………………………………………………………………………………………………………………………………………………………………

1. In the first month, how often did you use the Garmin device:

Everyday ☐ A few times per week ☐ Once a week ☐ Infrequently ☐

1. On average, in the past 3 months, on how many days each week did you use the Garmin device:

Everyday ☐ 6 days per week ☐ 5 days per week ☐ 4 days per week ☐

3 days per week ☐ 2 days per week ☐ 1 day per week ☐

1. On a scale of 1 to 5, (1 being not at all useful, 5 being extremely useful) how useful was the Garmin device for reminding you to break up your sitting?

1 2 3 4 5

☐ ☐ ☐ ☐ ☐

1. The Garmin device encouraged me to reduce the time I spend sitting
   1. Strongly agree ☐
   2. Agree ☐
   3. Neither agree or disagree ☐
   4. Disagree ☐
   5. Strongly disagree ☐
2. Do you have any other comments about the Garmin device? (e.g., usefulness, good points, improvements needed)

……………………………………………………………………………………………………………………………………………………………………………………………………………………………………………………………………………………………………………………………………………………………………………………………………………………………………………………………………………………………………………………………………………………………………………………………………………………………..

**Section 3: Frail-LESS peer support group feedback**

You were offered the opportunity to be in a Frail-LESS peer support group.

1. Did you engage with the Frail-LESS support group? Yes ☐ No ☐

If **YES go to Q2**, if **NO** please use this space to tell us the reasons why you did not engage with the group

………………………………………………………………………………………………………………………………………………………………………………………………………………………………………………………………………………………………………………………………………………………………………………………………………………………………………………………………………

1. Did you attend any of the peer support group sessions? Yes ☐ No ☐

If **YES**, please tell us what your experiences were of the group sessions.

………………………………………………………………………………………………………………………………………………………………………………………………………………………………………………………………………………………………………………………………………………………………………………………………………………………………………………………………………

1. On average, how often did you communicate with other people in the support group:

Everyday ☐ A few times per week ☐ Once a week ☐ Infrequently ☐

1. On a scale of 1 to 5, (1 being not at all useful, 5 being extremely useful) how useful did you find the support group for helping you to reduce your sitting?

1 2 3 4 5

☐ ☐ ☐ ☐ ☐

1. The peer support group helped encourage me to reduce the time I spend sitting
   1. Strongly agree ☐
   2. Agree ☐
   3. Neither agree or disagree ☐
   4. Disagree ☐
   5. Strongly disagree ☐
2. Do you have any other comments about the support group? (e.g., usefulness, good points, improvements needed)

……………………………………………………………………………………………………………………………………………………………………………………………………………………………………………………………………………………………………………………………………………………………………………………………………………………………………………………………………………………………………………………………………………………………………………………………………………………………..

**Section 4: Health coaching**

You were offered support sessions with a health coach.

1. Did you make use of the health coach sessions? Yes ☐ No ☐

If **YES go to Q2**, if **NO** please use this space to tell us the reasons why you did not use it

………………………………………………………………………………………………………………………………………………………………………………………………………………………………………………………………………………………………………………………………………………………………………………………………………………………………………………………………………

1. How many sessions have you had with a health coach:

One ☐ Two ☐ Three ☐ Four ☐ Five ☐

1. On a scale of 1 to 5, (1 being not at all useful, 5 being extremely useful) how useful did you find the health coaching sessions for helping you to reduce your sitting?

1 2 3 4 5

☐ ☐ ☐ ☐ ☐

1. The health coaching sessions helped encourage me to reduce the time I spend sitting
   1. Strongly agree ☐
   2. Agree ☐
   3. Neither agree or disagree ☐
   4. Disagree ☐
   5. Strongly disagree ☐
2. Do you have any other comments about the health coaching? (e.g., usefulness, good points, improvements needed)

……………………………………………………………………………………………………………………………………………………………………………………………………………………………………………………………………………………………………………………………………………………………………………………………………………………………………………………………………………………………………………………………………………………………………………………………………………………………..

**Section 5: Sitting and activity feedback sheet**

As part of the Frail-LESS programme, you were provided with a feedback sheet on your sitting, standing and stepping measurements that we took from you.

1. Did you make use of the feedback sheet? Yes ☐ No ☐

If **YES go to Q2**, if **NO** please use this space to tell us the reasons why you did not use it

………………………………………………………………………………………………………………………………………………………………………………………………………………………………………………………………………………………………………………………………………………………………………………………………………………………………………………………………………

1. On a scale of 1 to 5, (1 being not at all useful, 5 being extremely useful) how useful did you find the feedback sheet for helping you to reduce and break up your sitting?

1 2 3 4 5

☐ ☐ ☐ ☐ ☐

1. The feedback helped encourage me to reduce the time I spend sitting
   1. Strongly agree ☐
   2. Agree ☐
   3. Neither agree or disagree ☐
   4. Disagree ☐
   5. Strongly disagree ☐
2. Do you have any other comments about the feedback sheet? (e.g., usefulness, good points, improvements needed)

……………………………………………………………………………………………………………………………………………………………………………………………………………………………………………………………………………………………………………………………………………………………………………………………………………………………………………………………………………………………………………………………………………………………………………………………………………………………..

**Section 6: Alternative Support**

1. Have you used any other devices/tools/methods to encourage you to reduce the time you spend sitting that weren’t suggested in the programme? Yes ☐ No ☐
2. If **YES** please use this space to tell us which tools/devices/methods you have used and how they have helped

………………………………………………………………………………………………………………………………………………………………………………………………………………………………………………………………………………………………………………………………………………………………………………………………………………………………………………………………………………………………………………………………………………………………………………………………………………………………

………………………………………………………………………………………………………………………………………………………………………………………………………………………………………………………………………………………………………………………………………………………………………………………………………………………………………………………………………

**Section 7: Strategies to sit less**

Please list all of the strategies you have used to sit less throughout your day and tick whether you did this at home or outside of the home or tick both

| **Strategy** | **At home** | **Outside of the home** |
| --- | --- | --- |
| 1. |  |  |
| 2. |  |  |
| 3. |  |  |
| 4. |  |  |
| 5. |  |  |
| 6. |  |  |
| 7. |  |  |
| 8. |  |  |
| 9. |  |  |
| 10. |  |  |

**Section 8: Things that get in the way of sitting less (i.e., barriers)**

Please list any barriers that you have or are experiencing when it comes to trying to reduce your sitting time:

1.……………………………………………………………………………………………………………………………………………………………………………………………………………………………………………………………………………………………………………2……………………………………………………………………………………………………………………………………………………

………………………………………………………………………………………………………………………………………………………

3……………………………………………………………………………………………………………………………………………………

………………………………………………………………………………………………………………………………………………………

4.……………………………………………………………………………………………………………………………………………………………………………………………………………………………………………………………………………………………………………5……………………………………………………………………………………………………………………………………………………

………………………………………………………………………………………………………………………………………………………

**Section 9: Other Behavioural Changes**

1. In the past 3 months has anything in your life changed that has had an impact on your health-related behaviours (e.g., moved house, joined a gym, started a diet group, had a major life event)?

Yes ☐ No ☐

If **YES,** could you please **briefly explain what changes and how** it has impacted your behaviours below:

………………………………………………………………………………………………………………………………………………………………………………………………………………………………………………………………………………………………………………………………………………………………………………………………………………………………………………………………………

………………………………………………………………………………………………………………………………………………………

………………………………………………………………………………………………………………………………………………………

**Section 10: Testing sessions**

The testing sessions that took place during the study are not a part of the Frail-LESS programme but are a way to find out how the programme affects health. We are, however, interested in finding out how these testing sessions may have affected your decisions to take part in the study or how they may have changed your lifestyle behaviours.

1. Please let us know about your perceptions of the testing session measurements you have received as part of the study:

|  | Strongly disagree | Disagree | Neither disagree or agree | Agree | Strongly Agree |
| --- | --- | --- | --- | --- | --- |
| The offer of the testing session measurements encouraged me to participate in the study |  |  |  |  |  |
| The testing sessions motivated me to want to change how much time **I spent sitting** |  |  |  |  |  |
| Knowing that I would receive a follow up testing session motivated me to want to change **aspects of my lifestyle behaviours** |  |  |  |  |  |
| Knowing that I would receive a follow up testing session motivated me to want to change how much time **I spent sitting** |  |  |  |  |  |

If you agreed or strongly agreed with the statements above, please tell us how the testing sessions impacted on your life and/or sitting behaviour:

………………………………………………………………………………………………………………………………………………………………………………………………………………………………………………………………………………………………………………………………………………………………………………………………………………………………………………………………………

Thank you for taking the time to give us your feedback on the Frail-LESS programme

**INTERVENTION EVALUATION – 6 MONTHS**

Frail-LESS study

**Section 1: Wearable device feedback**

You were provided with a Garmin Vivofit wearable device to help you to track your sitting time and provide prompts to break up sitting.

1. Did you use the Garmin device given to you? Yes ☐ No ☐

If **YES go to Q2**, if **NO** please use this space to tell us the reasons why you did not use it

………………………………………………………………………………………………………………………………………………………………………………………………………………………………………………………………………………………………………………………………………………………………………………………………………………………………………………………………………

1. On average, in the past 3 months, on how many days each week did you use the Garmin device:

Everyday ☐ 6 days per week ☐ 5 days per week ☐ 4 days per week ☐

3 days per week ☐ 2 days per week ☐ 1 day per week ☐

1. On a scale of 1 to 5, (1 being not at all useful, 5 being extremely useful) how useful was the Garmin device for reminding you to break up your sitting?

1 2 3 4 5

☐ ☐ ☐ ☐ ☐

1. The Garmin device encouraged me to reduce the time I spend sitting
   1. Strongly agree ☐
   2. Agree ☐
   3. Neither agree or disagree ☐
   4. Disagree ☐
   5. Strongly disagree ☐
2. Do you have any other comments about the Garmin device? (e.g., usefulness, good points, improvements needed)

……………………………………………………………………………………………………………………………………………………………………………………………………………………………………………………………………………………………………………………………………………………………………………………………………………………………………………………………………………………………………………………………………………………………………………………………………………………………..

**Section 2: Frail-LESS peer support group feedback**

You were offered the opportunity to be in a Frail-LESS peer support group.

1. Did you engage with the Frail-LESS support group? Yes ☐ No ☐

If **YES go to Q2**, if **NO** please use this space to tell us the reasons why you did not engage with the group

………………………………………………………………………………………………………………………………………………………………………………………………………………………………………………………………………………………………………………………………………………………………………………………………………………………………………………………………………

1. Did you attend any of the peer support group sessions? Yes ☐ No ☐

If **YES**, please tell us what your experiences were of the group sessions.

………………………………………………………………………………………………………………………………………………………………………………………………………………………………………………………………………………………………………………………………………………………………………………………………………………………………………………………………………

1. In the past 3 months, how often did you communicate with other people in the support group:

Everyday ☐ A few times per week ☐ Once a week ☐ Infrequently ☐

1. In the first month, how often did you communicate with other people in the support group:

Everyday ☐ A few times per week ☐ Once a week ☐ Infrequently ☐

1. On a scale of 1 to 5, (1 being not at all useful, 5 being extremely useful) how useful did you find the support group for helping you to reduce your sitting?

1 2 3 4 5

☐ ☐ ☐ ☐ ☐

1. The peer support group helped encourage me to reduce the time I spend sitting
   1. Strongly agree ☐
   2. Agree ☐
   3. Neither agree or disagree ☐
   4. Disagree ☐
   5. Strongly disagree ☐
2. Do you have any other comments about the support group? (e.g., usefulness, good points, improvements needed)

……………………………………………………………………………………………………………………………………………………………………………………………………………………………………………………………………………………………………………………………………………………………………………………………………………………………………………………………………………………………………………………………………………………………………………………………………………………………..

**Section 3: Health coaching feedback**

You were offered support sessions with a health coach.

1. Did you make use of the health coach sessions? Yes ☐ No ☐

If **YES go to Q2**, if **NO** please use this space to tell us the reasons why you did not use these sessions

………………………………………………………………………………………………………………………………………………………………………………………………………………………………………………………………………………………………………………………………………………………………………………………………………………………………………………………………………

1. How many sessions have you had with a health coach:

One ☐ Two ☐ Three ☐ Four ☐ Five ☐

1. On a scale of 1 to 5, (1 being not at all useful, 5 being extremely useful) how useful did you find the health coaching sessions for helping you to reduce your sitting?

1 2 3 4 5

☐ ☐ ☐ ☐ ☐

1. The health coaching sessions helped encourage me to reduce the time I spend sitting
   1. Strongly agree ☐
   2. Agree ☐
   3. Neither agree or disagree ☐
   4. Disagree ☐
   5. Strongly disagree ☐
2. Do you have any other comments about the health coaching? (e.g., usefulness, good points, improvements needed)

……………………………………………………………………………………………………………………………………………………………………………………………………………………………………………………………………………………………………………………………………………………………………………………………………………………………………………………………………………………………………………………………………………………………………………………………………………………………..

**Section 4: Sitting and activity feedback sheets**

As part of the Frail-LESS programme, you were provided with feedback sheets on your sitting, standing and stepping measurements that we took from you.

1. Did you make use of the feedback sheets? Yes ☐ No ☐

If **YES go to Q2**, if **NO** please use this space to tell us the reasons why you did not use them

………………………………………………………………………………………………………………………………………………………………………………………………………………………………………………………………………………………………………………………………………………………………………………………………………………………………………………………………………

1. On a scale of 1 to 5, (1 being not at all useful, 5 being extremely useful) how useful did you find the feedback sheets for helping you to reduce and break up your sitting?

1 2 3 4 5

☐ ☐ ☐ ☐ ☐

1. The feedback helped encourage me to reduce the time I spend sitting
   1. Strongly agree ☐
   2. Agree ☐
   3. Neither agree or disagree ☐
   4. Disagree ☐
   5. Strongly disagree ☐
2. Do you have any other comments about the feedback sheets? (e.g., usefulness, good points, improvements needed)

……………………………………………………………………………………………………………………………………………………………………………………………………………………………………………………………………………………………………………………………………………………………………………………………………………………………………………………………………………………………………………………………………………………………………………………………………………………………..

**Section 5: Alternative Support**

1. Have you used any other devices/tools/methods to encourage you to reduce the time you spend sitting that weren’t suggested in the programme? Yes ☐ No ☐
2. If **YES** please use this space to tell us which tools/devices/methods you have used and how they have helped

………………………………………………………………………………………………………………………………………………………………………………………………………………………………………………………………………………………………………………………………………………………………………………………………………………………………………………………………………………………………………………………………………………………………………………………………………………………………

………………………………………………………………………………………………………………………………………………………………………………………………………………………………………………………………………………………………………………………………………………………………………………………………………………………………………………………………………

**Section 6: Strategies to sit less**

Please list all of the strategies you have used to sit less throughout your day and tick whether you did this at home or outside of the home or tick both

| **Strategy** | **At home** | **Outside of the home** |
| --- | --- | --- |
| 1. |  |  |
| 2. |  |  |
| 3. |  |  |
| 4. |  |  |
| 5. |  |  |
| 6. |  |  |
| 7. |  |  |
| 8. |  |  |
| 9. |  |  |
| 10. |  |  |

**Section 7: Things that get in the way of sitting less (i.e., barriers)**

Please list any barriers that you have or are experiencing when it comes to trying to reduce your sitting time:

1.……………………………………………………………………………………………………………………………………………………………………………………………………………………………………………………………………………………………………………2……………………………………………………………………………………………………………………………………………………

………………………………………………………………………………………………………………………………………………………

3……………………………………………………………………………………………………………………………………………………

………………………………………………………………………………………………………………………………………………………

4.……………………………………………………………………………………………………………………………………………………………………………………………………………………………………………………………………………………………………………5……………………………………………………………………………………………………………………………………………………

………………………………………………………………………………………………………………………………………………………

**Section 8: Other Behavioural Changes**

1. In the past 3 months has anything in your life changed that has had an impact on your health-related behaviours (e.g., moved house, joined a gym, started a diet group, had a major life event)?

Yes ☐ No ☐

If **YES,** could you please **briefly explain what changes and how** it has impacted your behaviours below:

………………………………………………………………………………………………………………………………………………………………………………………………………………………………………………………………………………………………………………………………………………………………………………………………………………………………………………………………………

………………………………………………………………………………………………………………………………………………………

………………………………………………………………………………………………………………………………………………………

**Section 9: Testing session**

The testing sessions that took place during the study are not a part of the Frail-LESS programme but are a way to find out how the programme affects health. We are, however, interested in finding out how these testing sessions may have affected your decisions to take part in the study or how they may have changed your lifestyle behaviours.

1. Please let us know about your perceptions of the testing session measurements you have received as part of the study:

|  | Strongly disagree | Disagree | Neither disagree or agree | Agree | Strongly Agree |
| --- | --- | --- | --- | --- | --- |
| The offer of the testing session measurements encouraged me to participate in the study |  |  |  |  |  |
| The testing sessions motivated me to want to change how much time **I spent sitting** |  |  |  |  |  |
| Knowing that I would receive a follow up testing session motivated me to want to change **aspects of my lifestyle behaviours** |  |  |  |  |  |
| Knowing that I would receive a follow up testing session motivated me to want to change how much time **I spent sitting** |  |  |  |  |  |

If you agreed or strongly agreed with the statements above, please tell us how the testing sessions impacted on your life and/or sitting behaviour:

………………………………………………………………………………………………………………………………………………………………………………………………………………………………………………………………………………………………………………………………………………………………………………………………………………………………………………………………………

Thank you for taking the time to give us your feedback on the Frail-LESS programme
