# Supplementary material for: Acceptability of a remotely delivered sedentary behaviour intervention to improve sarcopenia and maintain independent living in older adults with frailty: a mixed-methods study
Source: BMC Geriatr. 2024 Oct 11;24:820. doi: 10.1186/s12877-024-05385-4 (PMC11468285; doi:10.1186/s12877-024-05385-4)
Supplement: Supplementary file 4 — Additional file 4. [file 12877_2024_5385_MOESM4_ESM.docx]

**PROCESS EVALUATION INTERVIEW SCHEDULE (INTERVENTION)**

*The aim of this discussion is to talk about your experiences of the Frail-LESS programme. We would like to know about your experiences of the study and how the study may or may not have changed your behaviour. The discussion will last for approximately 45-60 minutes. There are no right or wrong answers, so please speak freely and honestly as we are interested in your own opinions and experiences. We are keen to understand what works and what needs improving.*

*Would it be OK if I record the conversations, this will just mean that I won’t have to write everything down and can listen to you with my full attention. The recording will only be listened to by the research team and will be transcribed and anonymised to ensure that you cannot be identified. Also, you can leave the interview at any time. Thank you.*

*I’ll just start with a few general questions and then ask you specifically about some of the components of the intervention/programme.*

***GENERAL QUESTIONS***

1. What motivated you to take part in the study?

The main aim of the study was to help older frail adults to reduce the time they spend sitting throughout the day.

1. What impact did the Frail-LESS programme have on your sitting behaviour?
2. How has your sitting behaviour changed?
   1. *Prompt: At work? At home? When outside the home?*
3. What motivated you to make these changes?
4. Do you think you would have made any of those changes without this programme?
   1. Why might this be?
5. What worked well to support reductions in sitting time during the programme?
   1. *Prompts: feedback on sitting, education session/workbook, wearable device, health coaching sessions, Frail-LESS support group.*
6. What could be improved to support reductions in sitting time?
7. Who knew you were involved in the study?
   1. *Prompt: Family? Friends? Work Colleagues?*
   2. How did they support you?
8. What should we consider for a future study that we haven’t covered?

***MEASUREMENT SESSIONS***

1. What do you remember about the measurements that we took as part of the study?
2. How did you feel when this data was collected?
3. What should we consider when taking these measures in a future study?
4. How did having the measures taken at the beginning impact on you?
   1. *Prompt:* *How did it influence your behaviour?*
   2. *Prompt: How about the follow up measurement sessions at 3 and 6 months?*

Ending Question: Is there anything that you would like to add?
