# Supplementary material for: Acceptability of a remotely delivered sedentary behaviour intervention to improve sarcopenia and maintain independent living in older adults with frailty: a mixed-methods study
Source: BMC Geriatr. 2024 Oct 11;24:820. doi: 10.1186/s12877-024-05385-4 (PMC11468285; doi:10.1186/s12877-024-05385-4)
Supplement: Supplementary file 5 — Additional file 5. [file 12877_2024_5385_MOESM5_ESM.docx]

**Additional File 5.** Engagement and experiences with the online peer support group and tailored activPAL feedback sheet

**Experiences with the online peer support group**

|  |  | **3-Months** | | **6-Months** | |
| --- | --- | --- | --- | --- | --- |
|  |  | n=11 | | n=12 | |
|  |  | n / mean | % / SD | n / mean | % / SD |
| On average, how often did you communicate with other people in the support group? | *Every day* | 0 | 0% | 0 | 0% |
|  | *A few times per week* | 0 | 0% | 0 | 0% |
|  | *Once a week* | 0 | 0% | 0 | 0% |
|  | *Infrequently* | 11 | 100% | 12 | 100% |
| How useful did you find the support group for helping you to reduce your sitting? (*5=Extremely useful, 1=Not at all useful)* | | 2.6 | 0.9 | 2.8 | 1.0 |
| The peer support group helped encourage me to reduce the time I spend sitting* | *Strongly agree* | 2 | 18% | 1 | 8% |
|  | *Agree* | 3 | 27% | 7 | 58% |
|  | *Neither agree nor disagree* | 3 | 27% | 2 | 17% |
|  | *Disagree* | 2 | 18% | 2 | 17% |
|  | *Strongly disagree* | 1 | 9% | 0 | 0% |

*This questionnaire item was only completed by participants who answered ‘Yes’ to attending the peer support group.

% calculated as number of responses / number of participants that fully completed this set of questionnaire items x 100.

**Experiences with the tailored activPAL feedback sheet**

|  |  | **3-Months** | | **6-Months** | |
| --- | --- | --- | --- | --- | --- |
|  |  | n=14 | | n=17 | |
|  |  | n / mean | % / SD | n / mean | % / SD |
| How useful did you find the feedback sheet for helping you to reduce and break up your sitting? (*5=Extremely useful, 1=Not at all useful)* | | 4.1 | 0.9 | 3.9 | 0.9 |
| The feedback helped encourage me to reduce the time I spend sitting | *Strongly agree* | 4 | 29% | 5 | 29% |
|  | *Agree* | 8 | 57% | 7 | 41% |
|  | *Neither agree nor disagree* | 2 | 14% | 4 | 24% |
|  | *Disagree* | 0 | 0% | 0 | 0% |
|  | *Strongly disagree* | 0 | 0% | 1 | 6% |

% calculated as number of responses / number of participants that fully completed this set of questionnaire items x 100.
